# Breastfeeding for 3 Months or Longer but Not Probiotics Is Associated with Reduced Risk for Inattention/Hyperactivity and Conduct Problems in Very-Low-Birth-Weight Children at Early Primary School Age

**DOI:** 10.3390/nu12113278

**Published:** 2020-10-26

**Authors:** Christoph Härtel, Juliane Spiegler, Ingmar Fortmann, Mariana Astiz, Henrik Oster, Bastian Siller, Dorothee Viemann, Thomas Keil, Tobias Banaschewski, Marcel Romanos, Egbert Herting, Wolfgang Göpel

**Affiliations:** 1 Department of Pediatrics, University Hospital of Würzburg, 97080 Würzburg, Germany; 2 Department of Pediatrics, University Hospital Schleswig-Holstein Campus Lübeck, 23538 Lübeck, Germany; matsingmar.fortmann@uksh.de (I.F.); bastian.siller@uksh.de (B.S.); Egbert.Herting@uksh.de (E.H.); wolfgang.goepel@uksh.de (W.G.); 3 Institute of Neurobiology, Center of Brain, Behavior and Metabolism, University of Lübeck, 23538 Lübeck, Germany; m.astiz@uni-luebeck.de (M.A.); henrik.oster@uni-luebeck.de (H.O.); 4 Department of Pediatric Pneumology, Allergy and Neonatology, Hannover Medical School, 30625 Hannover, Germany; viemann.dorothee@mh-hannover.de; 5 Institute for Clinical Epidemiology and Biometry, University of Würzburg, 97080 Würzburg, Germany; Thomas.Keil@lgl.bayern.de; 6 State Institute of Health, Bavarian Health and Food Safety Authority, 97688 Bad Kissingen, Germany; 7 Institute of Social Medicine, Epidemiology and Health Economics, Charité – Universitätsmedizin Berlin, 10117 Berlin, Germany; 8 Department of Child and Adolescent Psychiatry and Psychotherapy, Central Institute of Mental Health, Medical Faculty Mannheim, University of Heidelberg, 68159 Mannheim, Germany; tobias.banaschewski@zi-mannheim.de; 9 Department of Child and Adolescent Psychiatry, Psychosomatics and Psychotherapy, Center of Mental Health, University Hospital of Würzburg, 97080 Würzburg, Germany; Romanos_M@ukw.de

**Keywords:** breastfeeding, probiotic prophylaxis, preterm children, strength and difficulties, inattention/hyperactivity, intelligence

## Abstract

(1) Background: We aimed to evaluate the effect of proposed “microbiome-stabilising interventions”, i.e., breastfeeding for ≥3 months and prophylactic use of *Lactobacillus acidophilus/ Bifidobacterium infantis* probiotics on neurocognitive and behavioral outcomes of very-low-birthweight (VLBW) children aged 5–6 years. (2) Methods: We performed a 5-year-follow-up assessment including a strength and difficulties questionnaire (SDQ) and an intelligence quotient (IQ) assessment using the Wechsler Preschool and Primary Scale of Intelligence (WPPSI)-III test in preterm children previously enrolled in the German Neonatal Network (GNN). The analysis was restricted to children exposed to antenatal corticosteroids and postnatal antibiotics. (3) Results: 2467 primary school-aged children fulfilled the inclusion criteria. In multivariable linear regression models breastfeeding ≥3 months was associated with lower conduct disorders (B (95% confidence intervals (CI)): −0.25 (−0.47 to −0.03)) and inattention/hyperactivity (−0.46 (−0.81 to −0.10)) as measured by SDQ. Probiotic treatment during the neonatal period had no effect on SDQ scores or intelligence. (4) Conclusions: Prolonged breastfeeding of highly vulnerable infants may promote their mental health later in childhood, particularly by reducing risk for inattention/hyperactivity and conduct disorders. Future studies need to disentangle the underlying mechanisms during a critical time frame of development.

## 1. Introduction

Very-low-birth-weight children (VLBWI) have shown an increased risk for development of neurocognitive and behavioral long-term problems such as attention-deficit disorder [1,2]. The underlying pathophysiology is not well understood. Endogenous factors such as brain immaturity and dysregulated immune responses leading to inflammation-mediated morbidity play a crucial role [3]. Environmental factors such as intensive care, stress, maternal separation, nutritional deficits, circadian rhythm disruption, antibiotics and steroids have also been associated with altered brain development and may therefore contribute to the risk profile of VLBW children [2,3,4,5,6,7]. All these factors have an impact on fragile immune–microbiome co-establishment in preterm children, which occurs in timely association with central nervous system (CNS) development. Hence the “gut–brain axis” has been proposed as an interesting target for neuroprotective strategies. Breastfeeding, specifically for longer than 3 months, is nature’s most powerful concept of prevention. Breastfeeding has several beneficial effects including nutrition and growth, fostering immune–microbiome interplay and promoting mother–child interaction as well as improved long-term neurobehavioral outcome [8,9,10]. Mothers of preterm children, however, are often in a vulnerable situation in which motherhood and breastfeeding begin in an unfamiliar and stressful setting [11,12]. Hence breastfeeding is often ceased earlier than desired [13]. For non-breastfed children treatment with probiotic bacteria that excel as gut colonizers of breast-milk-fed children [14] could provide an alternative option to stabilize immune–microbiome co-development [15,16,17]. Recent animal studies provide some evidence indicating that probiotics may reverse effects of early-life stress and maternal separation on neural circuits underpinning fear expression and extinction [18]; however, there is no translational study in the setting of vulnerable children yet.

We performed a longitudinal study of VLBW children who were treated with antenatal steroids and postnatal antibiotics. These drugs induce early distortions of immune–microbiome co-development, e.g., gut dysbiosis, which has potential impact on neurocognitive and behavioral outcome. The main objective of this study was to determine the effect of breastfeeding for ≥3 months and/or prophylactic treatment of *Lactobacillus acidophilus/Bifidobacterium infantis* probiotics as proposed “microbiome-stabilizing” interventions on parent-rated behavioral outcome and neurobehavioral outcome manifest in IQ at early primary school age.

## 2. Patients and Methods

### 2.1. Cohort

The German Neonatal Network (GNN) is a population-based observational multicenter cohort study enrolling VLBWI (birth weight < 1500 g; gestational age of 22 + 0 ≤ 36 + 6 weeks) at 68 neonatal intensive care units (NICU) in Germany. Within the study, period data were collected from children born between 1 January 2009 and 31 December 2014 who had a standardized follow-up assessment by a constant study team consisting of a physician and two nurses from the leading study site in Lübeck at early primary school age (5–6 years old). Follow-up capacity is about 25–30% of those VLBWI included after birth. After obtaining written informed parental consent, predefined data regarding general neonatal information, antenatal and postnatal treatment and outcome were recorded for each patient on clinical record files at the participating centers. After discharge, data sheets were sent to the study center (University of Lübeck). Data quality was evaluated by a physician trained in neonatology via annual on-site monitoring of completed record files. After monitoring, data were coded and evaluated. For the purpose of our analysis, we included children who had been treated with any dose of antenatal steroids and any dose of postnatal antibiotics irrespective of the time point of treatment.

### 2.2. Variables

#### 2.2.1. Primary Outcome

To assess parent-rated behavior of their children, we used a strength and difficulties questionnaire (SDQ) [19]. The SDQ consists of 25 items comprising five subscales measuring hyperactivity inattention (e.g., restless, overactive and cannot stay still for long), emotional symptoms (e.g., many fears and easily scared), conduct problems (e.g., often lies or cheats), relationship with peers (e.g., picked on and bullied by other children), and prosocial behavior (e.g., considerate of other people’s feelings) that are scored on a 3-point Likert-type scale: 0 = not true, 1 = somewhat true and 2 = certainly true. There were 20 questions from 4 subscales related to problems that were used to calculate the total SDQ score (range 0–40) with higher scores indicating a worse outcome. A total SDQ score of 16 or higher using German norm values [20] is classified as having impaired mental health.

Subscale prosocial behavior was assessed with 5 questions (subscore range 0–10), and higher scores indicate more prosocial behavior.

Intelligence quotient (IQ) was assessed using the Wechsler Preschool and Primary Scale of Intelligence—Third Edition (WPPSI-III, German). The test represents intellectual functioning in verbal and performance cognitive domains and provides a composite score for general intellectual ability (i.e., full-scale IQ as applied herein). A higher score indicates higher intelligence.

#### 2.2.2. Main Treatments

Breastfeeding duration in months—defined as either exclusive breastfeeding (continuous breastfeeding) or a mix of breastfeeding and bottle feeding with expressed breast milk or formula—was assessed by a questionnaire at the age of 5–6 years, asking whether the child was breastfed and for how long (in months, ranging from 0–15 months). Breastfeeding ≥3 months was used as the cut-off as previously described [21]. This time frame is considered as an adequate period according to guidelines on allergy prevention and nutrition and on “imprinting” of immune–microbiome interaction.

Probiotic treatment: the probiotic formulation consisting of *B. infantis.* and *L. acidophilus* has been mostly used among the participating study sites [22]. Probiotics were administered once daily in single-dose capsules beginning from days 1–3 of life until days 14–35 of life. Each dose contained 1–3 × 10^9^ CFU *L. acidophilus* and 1–1.5 × 10^9^ *B. infantis*.

#### 2.2.3. Control Variables

Motor impairment (MI) was defined as a doctor-given diagnosis of cerebral palsy or functional deficits in analogy to the Gross Motor Function Classification System (GMFCS [23]) above or equal to level 1 or the Bimanual Fine Motor Function (BFMF [24]) above or equal to level 1.

Gestational age (GA) was calculated from the best obstetric estimate based on early prenatal ultrasound and obstetric examination.

Small-for-gestational age (SGA) was defined as a birth weight below the 10th centile [25] for gestational age according to gender-specific standards for birth weight by gestational age in Germany.

Bronchopulmonary dysplasia (BPD) was diagnosed as need of oxygen or ventilatory support at 36 weeks of gestational age.

Multiple birth was documented from medical records and data were included in the analysis and entered as a confounder in regression analysis.

Maternal age (in years) was taken from medical records after birth.

Maternal educational level was assessed by a questionnaire at 5–6 years and coded as low (compulsory school education or less), medium (International Classification of Education (ISCED) [26] level 2) or high (ISCED level 3 or above).

### 2.3. Statistical Analyzes

Data analyses were performed using the SPSS 26.0 data analysis package (IBM, Armonk, New York, NY, USA). Descriptive data for participant and non-participants and participants with and without breastfeeding ≥3 months were compared using chi-square test for categorical variables and t-test for continuous variables (Table 1). A *p*-value < 0.05 was considered statistically significant.

Linear regression models were performed with SDQ total score, subscores and IQ as dependent variables and potential “microbiome-stabilising” elements such as breastfeeding or probiotic treatment as independent variables (Model 1, unadjusted for confounding factors). Model 2 was adjusted for probiotics treatment (in analysis of breastfeeding), breastfeeding (in analysis of probiotic treatment) and known potential confounders for neurocognitive and behavioral outcome, specifically maternal educational level but also gestational age and gender [9]. Model 3 included factors of model 2 and was additionally adjusted for somatic disorders such as BPD and cerebral palsy (motor impairment), which were significantly different between participants with and without breastfeeding ≥3 months in univariate analyses (Table 1) and other supposed confounders of neurobehavioral outcome based on literature (SGA, multiple birth and maternal age, Table 2 and Table A1). Data are given as regression-coefficient B and 95% confidence intervals. To determine the relationship of breastfeeding ≥3 months and probiotics, we compared possible combinations of both parameters (interaction term, Table A2). Effect size and 95% confidence intervals (CI) were calculated.

### 2.4. Ethical Approval

All study parts were ethically approved by the University of Lübeck Ethical Committee and the committees of the participating centers (vote no. 08-022). Informed consent was obtained from all subjects. All methods were carried out in accordance with relevant guidelines and regulations, specifically the Declaration of Helsinki, the current revision of International Council for Harmonization (ICH) Topic E6, the Guidelines for Good Clinical Practice, the Guidelines of the Council for the International Organization of Medical Sciences and the WHO (“Proposed International Guidelines for Biomedical Research Involving Human Subjects”).

## 3. Results

**Figure A1:**
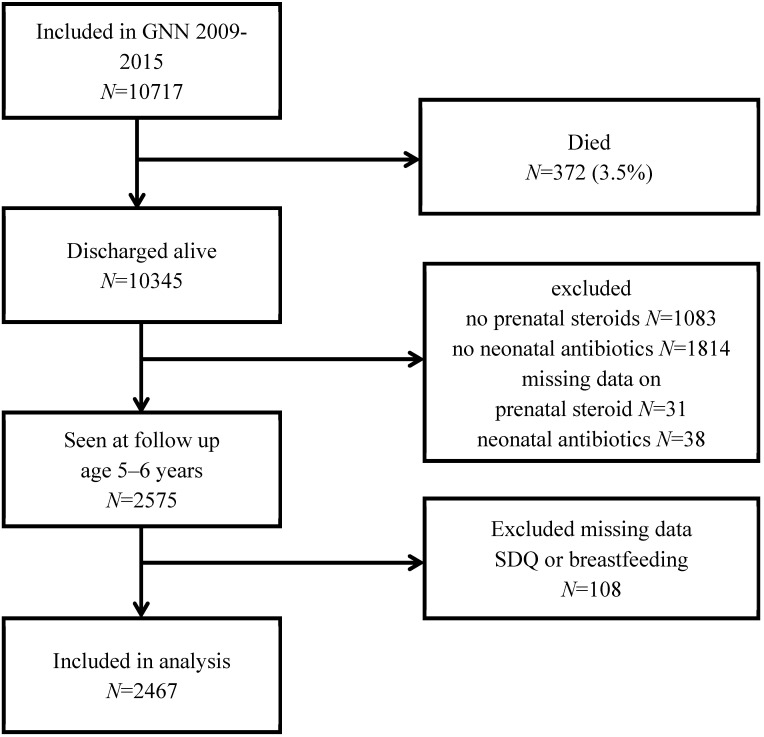
Flow chart of study participation.

A total of 10,345 VLBWI included between 2009 and 2013 were discharged from primary stay in hospital alive. Of these VLBWI, we excluded 1083 who received no prenatal steroids, 31 with missing data on prenatal steroids, 1814 who received no neonatal antibiotics and 38 with missing data of postnatal antibiotic therapy. Those VLBWI excluded were born more mature and suffered less neonatal complications. The 2575 VLBW children meeting the inclusion criteria were followed up at 5–6 years. Of these, 108 children were excluded due to missing data on either breastfeeding or SDQ (flow chart, Figure A1). The study cohort consisted of 2467 children with a median gestational age at birth of 27.9 (26.1–29.4), a median birth weight of 980 g (745–1220 g) and a high rate of multiples (39.9%). Of these children, 358 (14.5%) had SDQ scores of 16 or higher, indicating impaired mental health. Descriptive data for those excluded and comparison of included children stratified for breastfeeding ≥3 months are shown in Table 1. The breastfeeding rate for ≥3 months was 88%.

### 3.1. Breastfeeding ≥3 months and Behavioral Outcome and IQ at Early Primary School Age

Breastfeeding ≥3 months was associated with fewer conduct problems and less hyperactivity/inattention as measured by SDQ subscores, even after controlling potential confounders and probiotic administration (Table 2 and Table A1). Adjustment for SGA, multiple births, maternal age, bronchopulmonary dysplasia (BPD) and motor impairment did not change the results compared to adjusting for known confounders (gestational age, sex and maternal education level).

Data on IQ scores at 5–6 years were available from 2126 children. Breastfeeding ≥3 months was associated with higher IQ values indicating higher assumed intelligence after adjusting for potential confounders including probiotic administration (Table 2 and Table A1). Sex and multiple births had no influence on the outcomes.

### 3.2. Neonatal Probiotic Treatment and Behavioral Outcome/IQ at Early Primary School Age

Data from 1851 VLBW children on prophylactic probiotics during the neonatal period were available. Probiotic treatment was not associated with any SDQ category or IQ (Table 2).

To address the relationship between breastfeeding ≥3 months and probiotic treatment we performed an adjusted linear regression analysis comparing combinations of treatments. Children who had received probiotics and were breastfed < 3 months had reduced scores for emotional problems but increased SDQ scores for inattention/hyperactivity problems (Table A2).

### 3.3. Long-Term Physical Impairments and Neurobehavioral Outcome at Early Primary School Age

Both motor impairment (MI) and BPD were associated with adverse behavioral outcome (higher SDQ total score, higher hyperactivity/inattention subscore, higher subscores for conduct problems and less prosocial behavior) and lower IQ (Table 2). No association of MI and BPD was noted with emotional symptoms and conduct problems (Table 2).

## 4. Discussion

We investigated risk factors for adverse behavioral outcome at 5–6 years of age in a cohort of 2467 highly vulnerable VLBW children with a history of antibiotic and steroid treatment during a critical period of neurodevelopment. Breastfeeding for ≥3 months was associated with lower parent-rated behavioral difficulty scores, particularly in the hyperactivity/attention deficit category, independent of maternal education levels. Full-scale IQ was higher in VLBW children who were breastfed ≥3 months. Chronic physical impairment such as BPD and motor impairment was also associated with adverse neurocognitive and behavioral outcomes. Considering the limitations of our observational study design, we found no benefit or harm of prophylactic use of *Lactobacillus acidophilus/Bifidobacterium infantis* probiotics in VLBW children on SDQ and IQ scores at early primary school age.

### 4.1. Breastfeeding and Behavioral Outcome and IQ at School Age

Data on the effect of breastfeeding and neuropsychiatric consequences are controversial. In line with our finding, a recent meta-analysis of mainly term-born children reported similar results [10]. This association has been described to be age dependent and ceases between the ages of 9 and 13 years [27]. However, we do not know whether all children equally benefit from breastfeeding regarding behavioral outcome. In contrast to our results, a large randomized breastfeeding intervention study with generally healthy children showed no effect of extended or exclusive breastfeeding on any SDQ scores at early primary school age [28]. Therefore, breastfeeding might be particularly (not specifically) beneficial for certain subgroups. Apart from the high-risk group of former VLBW preterm infants described in our study, breastfeeding has been discussed as a modulator in children with a high genetical risk for behavioral problems [29].

The benefit of breastfeeding on neurobehavioral outcomes has multifactorial aspects. First, human milk feeding of preterm babies as a nutritional source is generally associated with reduced short- and long-term morbidity [30,31,32]. One proposed mechanism is the stabilization of early immune–microbiome interaction by specific human milk abundant molecules such as alarmin S100 A8/A9. The latter has a pleiotropic effect on innate immunity including the prevention of gut dysbiosis and (sustained) inflammation [33,34,35]. A potential hallmark of children with hyperactivity/attention deficit problems is “mild inflammation”, as characterized by increased interferon-gamma blood levels [36]. Breastfeeding may induce a shift in cytokine patterns to a less proinflammatory cytokine signature which might (in)directly promote attention in vulnerable children [37]. Second, human milk exclusively contains nutrients such as polyunsaturated fatty acids (PUFA), docosahexaenoic acid (DHA) and arachidonic acid (ARA), which are important for neurocognitive development and myelination [32,38]. Third, breastfeeding has a strong socio-emotional component, which may prime behavior during a critical period of development. Milk release is mediated via oxytocin expression, which is induced by touch, warmth and eye contact during interaction between mother and child. Hence breastfeeding is proposed to promote the infant’s social perception, attention to positive emotional expressions, bonding and prosocial behavior and has anxiolytic effects [39]. The impact of breastfeeding on development of hyperactivity and attention deficit disorder has been widely researched and is still a current topic of interest. Julvez et al. [21] found an association between breastfeeding > 12 weeks and a lower rate of inattention and hyperactivity in two small population-based birth cohorts in Spain. Park et al. [40] noted an association between breastfeeding and behavioral problems after adjusting for children’s intelligence. In a case-control study of attention deficit/hyperactivity disorder (ADHD) in children aged 7–13 years, Stadler [41] demonstrated that it was not initiation of breastfeeding but rather duration of breastfeeding that was associated with ADHD risk. Adesman et al. [42] analyzed data from a nationally representative survey of children in the USA including parent-reported feeding history in the first 12 months and ADHD risk. There was a 5.5–10-fold increase in adjusted odds of preschool ADHD being identified in the exclusively formula-fed group compared with the exclusively breastfed group. In addition, duration of breastfeeding was crucial, suggesting that protective neurodevelopmental benefits of breastfeeding exist along a continuum, with lower risks of later ADHD for each additional month a mother continues breastfeeding [32,38].

### 4.2. Probiotic Treatment and Neurocognitive and Behavioral Outcome at School Age

To our knowledge, we describe the first large-scale study of behavioral data after prophylactic probiotic administration during the neonatal period. Probiotic treatment had no major impact on neurocognitive and behavioral outcome. In a comparison of small subgroups, probiotic treatment was potentially associated with lower emotional symptoms in those children who were not breastfed ≥3 months. A similar association was noted in healthy women who took fermented products with *Bifidobacteria*/*Lactobacilli*/*Lactococci* for 4 weeks and showed an activation of brain regions that control central processing of emotions [43]. We assume that probiotic treatment seems to have a weaker or no effect on the gut–brain axis in children who already receive “natural pro/pre/synbiotics” in the form of breastmilk. Further observational studies are needed to consolidate the hypothesis that the subgroup of children whose mothers were not able to breastfeed for 3 months may benefit from longer probiotic prophylaxis after cessation of breastfeeding to prevent emotional problems (and whether more inattention problems are of concern). Given the high-risk population in our study, human milk feeding and probiotics might be able to “reverse” antibiotic-induced gut dysbiosis [44], which also requires further long-term studies of extremely preterm children.

### 4.3. The Pivotal Interface Between Somatic and Mental Health

Somatic health issues such as chronic lung disease (BPD) or motor impairment and their relationship towards behavioral problems have rarely been studied in preterm born children [45]. In VLBW children a high correlation between cerebral palsy, intellectual disability and behavior problems has been described [46], and most studies exclude VLBW children with motor or sensory impairment. While breastfeeding did not influence motor development [47], breastmilk was shown to reduce incidence of BPD [30]. Additionally, BPD was shown to negatively influence cognitive development [48] and behavior [49]. In our study, the association of breastfeeding and less hyperactivity/inattention, conduct problems and higher IQ remained significant after controlling for BPD and motor impairment. We hypothesize that a pivotal interface between somatic and mental health issues exist, specifically for at-risk children. Our data underpin the need to elucidate common developmental trajectories of long-term physical and mental health, including risk and resilience signatures of immune–microbiome co-development (and its beneficial modulation).

#### Strengths and Limitations

The main strength of our investigation is the large population-based longitudinal design of the GNN study. The 68 participating sites care for about one third of all VLBWI born in Germany and had an enrolment rate of >70% of eligible children. Within this cohort, we analyzed a high-risk group with partial homogenization of risk patterns at the host–microbiome interface, i.e., inclusion criteria were exposure to antibiotics and antenatal steroids. Due to the prospective study design, we were able to control numerous confounders. We had a low proportion of missing data due to a study design with annual on-site monitoring of data. Probiotic treatment was systematically inquired since 2011, explaining the rather high rate of missing values. Of those VLBW children seen at ages 5–6 years, less than 5% were excluded due to missing data. The breastfeeding rate ≥3 months in our cohort is high (88%), which relates to the breastfeeding promotion strategy in many GNN centers. It introduces an imbalance in group sizes which we addressed with multivariate regression analyses. Center-specific effects were noted (data not shown) and cannot be fully adjusted for. In addition, we randomly invited more immature infants for standardized follow-up by one observer team. This may have introduced a selection bias. Our observational cohort study is mainly hypothesis-generating and has limitations that require cautious interpretation of our findings. First, parent-reported breastfeeding duration was assessed at the age of 5 years, which might limit an accurate recall. In a subgroup of parents breastfeeding duration was already inquired during the first year of life and correlation between recall during the first and sixth year of life was moderate (Pearson’s coefficient, 0.34). Second, for the association of breastfeeding and behavioral outcomes in generally healthy children there is a high number of potentially confounding factors that may explain many of the reported effects of breastfeeding on neurodevelopment [50]. Family disposition for allergies may have an impact on duration of breastfeeding. We controlled for a number of important socio-economic and neonatal factors; however, any observational study is by design limited in controlling for all possible confounders, and multicollinearity of predictors (in regression models) might exist. In line with this, maternal characteristics may affect whether a child is breastfed or not. Specifically, maternal psychopathology and maternal stress may impair the mother’s capacity or physical fitness to breastfeed the infant. Furthermore, psychiatric disorders such as ADHD are associated to a wide range of somatic disorders, potentially limiting the extent of breastfeeding [51,52]. Thus, future studies need to systematically acquire information about maternal and paternal psychopathology. In addition, children with hyperactivity/inattention may have been overtly hyperactive or inattentive during early infancy and, therefore, could not co-operate with the breastfeeding process over a sustained period of time. This may have led to a scenario where the duration of breastfeeding was already directly affected by presentation of early hyperactivity/inattention traits. Finally, age might be an important confounder for an association between breastfeeding and behavior. Influence of neonatal characteristics, complications and interventions (e.g., breastfeeding and probiotic treatment) on behaviour outcome might become smaller over time, while environmental influences (parent and peer support) become more important.

## 5. Conclusions

VLBW children are at high risk for development of neurobehavioral problems. Breastfeeding ≥3 months may promote mental health in these vulnerable children, particularly by reducing risk for inattention/hyperactivity and conduct problems. Proposing a pivotal interaction between chronic physical impairments and neurobehavioral outcome, breastfeeding ≥3 months has important preventive aspects of common trajectories of long-term child development. Translational studies need to further disentangle the underlying mechanisms, e.g., strengthening the mother–child interaction and fostering immune–microbiome co-development during a critical time frame. In contrast, probiotics during the neonatal period had no independent effect on behavioral outcome and intelligence of VLBW children at early primary school age.

## Figures and Tables

**Table 1:** Characteristics of very-low-birth-weight children (VLBWI) included after birth and those with follow-up included in the analysis.

		Excluded	Included in Analysis		
		*N* = 8250	Breastfeeding < 3 month *N* = 298	Breastfeeding ≥ 3 month *N* = 2169	*p*-Value
Gestational age (weeks)		29.1 (26.8–31.0) ^#^	28.1 (26.0–29.4)	27.9 (26.1–29.4)	n.s.
Female gender		4043/8250 (49.0%)	138/298 (46.3%)	1034/2169 (47.7%)	n.s.
Multiple		2707/10717 (32.8%) ^#^	116/298 (38.9%)	869/2169 (40.1%)	n.s.
Birth weight		1125 (840–1350) ^#^	960 (730–1210)	980 (745–1220)	n.s.
SGA		1616/8250 (19.6%) ^#^	50/298 (16.8%)	296/2169 (13.6%)	n.s.
BPD		1269/8246 (15.4%) ^#^	82/298 (27.5%)	438/2169 (20.2%)	0.004
IVH		1373/8238 (16.7%) ^#^	48/298 (16.1%)	407/2169 (18.8%)	n.s.
Probiotics		4787/6776 (70.3%) ^#^	174/226 (77%)	1218/1625 (75%)	n.s.
Maternal age		31 (27–35) ^#^	31 (27–35)	31 (28–32)	0.04
Motor impairment			98/298 (32.9%)	569/2169 (26.2%)	0.015
Maternal education	Low		93/278 (33.5%)	280/2096 (13.4%)	<0.001
	Medium		109/278 (39.2%)	714/2096 (34.1%)	
	High		76/278 (27.3%)	1102/2096 (52.6%)	
SDQ total score			10 (6–15)	9 (5–13)	<0.001
SDQ inattention/hyperactivity			5 (3–7)	4 (2–5)	<0.001

SGA: small-for-gestational age (<10th centile for gestational age), BPD: bronchopulmonary dysplasia (supplemental oxygen or ventilatory support at 36 weeks postmenstrual age), IVH: any intraventricular hemorrhage and SDQ: strength and difficulties questionnaire. Absolute numbers and percentages are given for categorical variables and medians (Q1–3) for continuous variables. The *p*-values (*t*-test for continuous variables and chi-square test for categorical variables) are given for comparison of children with/without breastfeeding > 3 months. ^#^: *p*-value < 0.05 for comparison of VLBWI included and excluded in the analysis. n.s.: not significant.

**Table 2:** Multivariable linear regression analysis for outcomes of the strength and difficulties questionnaire (SDQ), total and subscores (higher values indicating worse outcomes) and intelligence quotient (IQ; higher values indicating more intelligence).

Outcome Treatments	SDQ Total Score B (95% CI)	Emotional Symptoms B (95% CI)	Conduct Problems B (95% CI)	Hyperactivity/Inattention B (95% CI)	Peer Relationship Problems B (95% CI)	Prosocial Behavior B (95% CI)	IQ B (95% CI)
Sex: female vs male	**−0.53 (−1.02–(−0.04))**	**0.40 (0.22–0.57)**	**−0.22 (−0.37–(−0.08))**	**−0.59 (−0.82–(−0.36))**	−0.12 (−0.28–0.05)	**0.57 (0.40–0.74)**	−0.13 (−1.27–1.00)
Gestational age (in weeks)	**−0.31 (−0.43–(−0.19))**	−0.02 (−0.06–0.02)	−0.01 (−0.04–0.03)	**−0.17 (−0.23–(−0.12))**	**−0.11 (−0.15–(−0.07))**	0.03 (−0.01–0.08)	**1.00 (0.73–1.28)**
High vs medium/low maternal education	**−0.82 (−1.16–(−0.48))**	**−0.13 (−0.25–(−0.004))**	**−0.28 (−0.38–(−0.18))**	**−0.36 (−0.52–(−0.20))**	−0.05 (−0.17–0.06)	0.07 (−0.05–0.19)	**4.31 (3.51–5.11)**
SGA (yes vs no)	**1.28 (0.55–2.01)**	0.14 (−0.12–0.4)	0.15 (−0.06–0.36)	**0.81 (0.47–1.15)**	0.18 (−0.06–0.42)	−0.17 (−0.43–0.19)	**−3.31 (−5.04–(−1.58)**
Multiples (yes vs no)	**−0.81 (−1.31–(−0.30))**	**−0.29 (−0.47–(−0.11))**	0.02 (−0.13–0.17)	**−0.45 (−0.68–(−0.21))**	−0.09 (−0.26–0.08)	−0.04 (−0.22–0.13)	−0.72 (−1.87–0.43)
Maternal age (in years)	**−0.08 (−0.13–(−0.04))**	**−0.03 (−0.05–(−0.02))**	−0.01 (0.02–0.01)	**−0.05 (−0.07–(−0.03))**	0.004 (−0.01–0.02)	−0.01 (−0.02–0.01)	0.15 (0.41–0.26)
BPD (yes vs no)	**1.31 (0.65–1.97)**	0.16 (−0.07–0.40)	0.07 (−0.13–0.26)	**0.64 (0.33–0.94)**	**0.45 (0.23–0.67)**	**−0.34 (−0.57–(−0.11))**	**−2.93 (−4.51–(−1.34))**
Motor impairment (yes vs no)	**1.31 (0.70–1.92)**	0.05 (−0.172–0.27)	0.01 (−0.17–0.19)	**0.49 (0.21–0.77)**	**0.76 (0.56–0.96)**	**−0.57 (−0.78–(−0.36))**	**−6.18 (−7.65–(−4.71))**
Probiotics (yes vs no)	0.12 (−0.45–0.69)	−0.10 (−0.31–0.10)	0.03 (−0.13–0.20)	0.05 (−0.21–0.31)	0.14 (−0.05–0.33)	−0.19 (−0.39–0.01)	0.94 (−0.37–2.26)
Breastfeeding ≥ 3 vs < 3 months	−0.58 (−1.35–0.18)	0.23 (−0.05–0.50)	**−0.25 (−0.47–(−0.03))**	**−0.46 (−0.81–(−0.10))**	−0.10 (−0.36–0.15)	0.07 (−0.20–0.34)	**2.20 (0.43–3.97)**

SGA: small for gestational age (i.e., < 10th centile for gestational age). BPD: bronchopulmonary dysplasia at 36 weeks of gestational age. Statistically significant regression coefficients were given in bold. B: regression-coefficient. CI: confidence intervals.

## Appendix A

**Table A1:** Unadjusted and adjusted linear regression analyzes for the outcomes of the strength and difficulties questionnaire (SDQ) total and subscores and the intelligence quotient (IQ), considering breastfeeding and probiotic therapy separately as main treatment.

	Model 1 Unadj. B (95% CI)	Model 2 Adj. B (95% CI)	Model 3 Adj. B (95% CI)
Breastfeeding ≥ 3 vs < 3 months			
Total SDQ score	**−1.55 (−2.22–(−0.88))**	−0.76 (−1.54−0.02)	−0.58 (−0.14–0.18)
Emotional symptoms	−0.01 (−0.23–0.23)	0.21 (−0.06–0.49)	0.23 (−0.05–0.50)
Conduct problems	**−0.44 (−0.63–(−0.25))**	**−0.26 (−0.48–(−0.04))**	**−0.25 (−0.47–(−0.03))**
Hyperactivity/inattention	**−0.82 (−0.14–(−0.51))**	**−0.53 (−0.90–(−0.17))**	**−0.46 (−0.81–(−0.10))**
Peer problems	**−0.29 (−0.51–(−0.07))**	−0.18 (−0.44–0.08)	−0.10 (−0.36–0.15)
Prosocial behaviour	**0.29 (0.06–0.52)**	0.13 (−0.14–0.40)	0.07 (−0.20–0.34)
Full-scale IQ	**5.43 (3.70–7.16)**	**2.72 (0.88–4.55)**	**2.20 (0.43–3.97)**
Probiotic treatment (yes vs no)			
Total SDQ score	0.24 (−0.34–0.83)	0.08 (−0.51–0.66)	0.12 (−0.45–0.69)
Emotional symptoms	−0.09 (−0.29–0.12)	−0.11 (−0.31–0.10)	−0.10 (−0.31–0.10)
Conduct problems	0.03 (−0.14–0.19)	0.03 (−0.14–0.20)	0.03 (−0.13–0.20)
Hyperactivity/inattention	0.11 (−0.16–0.39)	0.03 (−0.24–0.30)	0.05 (−0.21–0.31)
Peer problems	0.18 (−0.01–0.38)	−0.18 (−0.44–0.08)	−0.10 (−0.36–0.15)
Prosocial behaviour	−0.16 (−0.36–0.04)	−0.18 (−0.39–0.02)	−0.19(−0.39–0.01)
Full-scale IQ	0.66 (−0.78–2.10)	1.02 (−0.35–2.38)	0.94 (−0.37–2.26)

Model 1 is unadjusted B (95% CI). Model 2 is adjusted for gestational age (weeks), gender and maternal education level. Model 3 is additionally adjusted for small for gestational age (<10th centile for gestational age), multiple birth, maternal age, diagnosis of bronchopulmonary dysplasia and motor impairment. Statistically significant regression coefficients were indicated in bold. B: regression-coefficient. CI: confidence intervals.

**Table A2:** Linear regression analysis for SDQ and IQ scores and subscores with combination of breastfeeding ≥3 months +/− probiotics as one main treatment.

	Breastfeeding and Probiotics *N* = 1218 B (95% CI)	Breastfeeding Only *N* = 407	Probiotics Only *N* = 174
SDQ, total score	0.06 (−1.5–1.6)	0.02 (−1.6–1.6)	0.8 (−0.9–2.5)
Emotional symptoms	−0.31 (−0.9–0.2)	−0.28 (−0.9–0.3)	**−0.68 (−1.3–(−0.01))**
Conduct problems	−0.04 (−0.5–0.4)	−0.05 (−0.5–0.4)	0.26 (−0.2–0.8)
Hyperactivity/inattention	0.16 (−0.5–0.9)	0.2 (−0.5–0.9)	**0.79 (0.01–1.6)**
Peer problems	0.26 (−0.3–0.8)	0.15 (−0.4–0.7)	0.43 (−0.1–1.0)
Prosocial behaviour	−0.19 (−0.7–0.4)	−0.02 (−0.6–0.5)	−0.29 (−0.9–0.3)
Full-scale IQ	1.9 (−1.6–5.4)	0.7 (−2.9–4.4)	−0.7 (−4.6–3.1)

A linear regression model (B (95% CI)) was applied including the combination term breastfeeding ≥3 months +/− probiotics as one main treatment in four different categories: breastfeeding ≥3 months + probiotics, breastfeeding ≥3 months only, probiotics but breastfeeding < 3 months and, as a reference category, no probiotics and breastfeeding < 3 months (*n* = 52), respectively. Within the model we adjusted for gestational age (weeks), gender, maternal education level, small for gestational age (<10th centile for gestational age), multiple birth, maternal age, diagnosis of bronchopulmonary dysplasia and motor impairment. Statistically significant regression coefficients were indicated in bold. SDQ: strength and difficulties questionnaire, IQ: Intelligence quotient. B: regression-coefficient. CI: confidence intervals.
